# Manipulation of Unknown Objects to Improve the Grasp Quality Using Tactile Information

**DOI:** 10.3390/s18051412

**Published:** 2018-05-03

**Authors:** Andrés Montaño, Raúl Suárez

**Affiliations:** Institute of Industrial and Control Engineering (IOC), Universitat Politècnica de Catalunya (UPC), 08028 Barcelona, Spain; andres.felipe.montano@upc.edu

**Keywords:** robotic manipulation, grasping, grasp quality, tactile sensors, tactile manipulation

## Abstract

This work presents a novel and simple approach in the area of manipulation of unknown objects considering both geometric and mechanical constraints of the robotic hand. Starting with an initial blind grasp, our method improves the grasp quality through manipulation considering the three common goals of the manipulation process: improving the hand configuration, the grasp quality and the object positioning, and, at the same time, prevents the object from falling. Tactile feedback is used to obtain local information of the contacts between the fingertips and the object, and no additional exteroceptive feedback sources are considered in the approach. The main novelty of this work lies in the fact that the grasp optimization is performed on-line as a reactive procedure using the tactile and kinematic information obtained during the manipulation. Experimental results are shown to illustrate the efficiency of the approach.

## 1. Introduction

Object manipulation is a common task in service and industrial robotics. The development of complex robotics hands has impulsed the search of manipulation strategies to take advantage of this hardware resource [1]. One of the common features of the new robotic hands is the inclusion of tactile sensors that allow to get information about the contacts with the manipulated object, increasing the robot capabilities. Usually, in a realistic scenario, the geometric model of the manipulated object is only partially known or even unknown. Tactile sensors help to recognize the manipulated object or to reduce the uncertainty in their geometric model.

The object manipulation process usually pursues three goals [2], either independently or in a combined way:

- From the hand point of view, the optimization of the hand configuration, i.e., searching for a particular hand configuration satisfying some specific constraints that can be arbitrarily defined.
- From the grasp point of view (relation hand-object), the optimization of the grasp quality, i.e., searching for a grasp that can resist external force perturbations on the object.
- From the object point of view, the optimization of the object configuration, i.e., searching for an appropriate object position and orientation that satisfy the requirements of a given task.

In order to manipulate an object, the first step is grasping it. Different grasp synthesis approaches have been proposed for known and unknown objects [3], but, in general, most of the grasp planners require an exact model of the object. Some approaches generate a set of feasible grasps and then choose the one that maximizes a quality metric [4,5,6], and others use a kinestatic formulation of the grasp synthesis problem considering simultaneously the grasping constraints [7]. Other works look for the grasping points on the object surface without considering the hand constraints, for instance, using geometric reasoning to find an optimal [8] or at least a valid grasps [9], or using an initial random grasp (that could not satisfy any quality criterion) to start a search of a valid or an optimal one either for single bodies [10] or for articulated objects [11]; these approaches require the evaluation of the grasp reachability for the used hand. Using tactile and visual feedback the planner can compute the grasp and adapt it to address problems such as slippage, the effect of external disturbances and, in some applications, the change of the grasped object weight [12]. When an exact object model is not available it can be approximated using geometric primitives [13] or learning methods can be applied to transfer a successful grasp of a known object to novel objects [14]. Uncertainty on the object shape has been modeled as constraints in the grasp planner [15], or as a noise managed using probabilistic techniques [16,17]. When the model of the object is completely unknown, a haptic exploration of the object surface can be performed prior to compute the grasp [18]. Other than the contact points, the execution of a grasp also requires the computation of proper grasping forces, which is another complex problem [19].

There are many quality indexes to evaluate the grasp quality [20,21]. One of the most used indexes is the measure of the largest perturbation wrench that the grasp can resist in any direction [22], but it does not consider the hand configuration. When the grasp can counterbalance a perturbation wrench in any direction, it is called a *force-closure* grasp (FC grasp) [23].

Tactile sensing systems based on different sensing techniques have been developed during the last decades in order to equip robots with tactile feedback [24,25]. Tactile feedback provides relevant information in many robotics applications [26]. In object manipulation, it reduces the uncertainty allowing, for instance, an improvement of the grasp stability and safety [27,28,29]. The tactile information obtained during the manipulation can also be used jointly with the hand kinematics to identify the model of the manipulated object [30], or jointly with visual feedback to improve the control performance [31].

Kinematics and control of multifingered hands manipulating an object with rolling contacts were already studied, but information about the mass, the center of mass and the geometry of the object is required [32]. On the other hand, different control strategies were proposed to deal with the manipulation of unknown objects, but tactile feedback is not always considered. A position-force control scheme was used to manipulate the object following a predefined trajectory [33], but it was evaluated only in simulation introducing noise on the sensor measurements to simulate a real environment. A torque controller was used to optimize the the applied grasping force over an object with smooth curvatures and a predefined shape [34], the approach can grasp objects with different shapes, but the experimental results were only performed in simulations without tactile sensors. A position-force controller was also used to slide the fingers on the object surface to explore and recognize it [35]. Another approach uses only a position control law to change the pose of the manipulated object [36], but it lacks of sensory feedback which is a hard limitation.

The manipulation space is the *n*-dimensional space defined by the values of all the finger joints, where a point represents a configuration of the hand and a curve represents a finger movement (i.e., a sequence of hand configurations). Then, doing a desired manipulation means following an appropriate curve in this space. However, computing a manipulation curve in advance may not be possible due to the unknown shape of the object, i.e., the manipulation constraints cannot be computed a priori and therefore planning a sequence of finger movements is not possible. In these conditions, manipulation must be a reactive procedure that determines on-line the proper hand movements. One straightforward way is the use of an exploration method [2] to search for hand configurations that improve a manipulation index, i.e., the fingers are moved following a predefine strategy and if the result improves the grasp (according to any quality index), a new step is done, otherwise the movement is drawn back and a new one is tested. In other words, it is like a blind search in the grasp space.

In this context, the main contributions of this work are: first, the proposal of a relationship between the finger joints and the manipulation indexes, i.e., the indexes are expressed as functions of the hand joint values, and second, a simple procedure to optimize the grasp of an unknown object by determining on-line the hand movements to manipulate the object following the gradient of these functions. As a result, with relatively simple geometrical reasoning and assumptions, an unknown object can be manipulated keeping the grasping forces in a desired range and preventing the object from falling despite uncertainty. It must be remarked that the expression “unknown object” means that the model of the object is not used at all in the manipulation procedure. Actually, as stated above, the shape of the object can be reconstructed using tactile and kinematics information during the manipulation [30]. These contributions make the approach presented in this work completely different from the approach presented in [2], where a blind search is performed to improve the grasp according to any index.

Tactile and kinematic data are inputs to the proposed manipulation process, which is a reactive procedure that controls locally the movements and contact forces to prevent the object from falling. The hand configuration is iteratively changed to manipulate the object optimizing three indexes associated with the three manipulation goals mentioned above, either individually or properly combined. Nevertheless, even when the computed movements should always improve the grasp quality, due to the unknown shape of the manipulated object and the different sources of noise and uncertainty, the actual grasp quality may eventually decrease in some manipulation steps.

The remaining of the paper is organized as follows. The proposed approach is detailed in Section 2. Section 3 introduces the three manipulation strategies to deal with each of the above mentioned manipulation goals. The experimental setup and results are presented in Section 4. Finally, some conclusions and future work are presented in Section 5.

## 2. Proposed Approach

### 2.1. Problem Statement, Approach Overview and Assumptions

The problem addressed in this work is the manipulation of unknown objects pursuing one or more of the manipulation goals mentioned in Section 1, i.e., optimizing the grasp from the point of view of the hand, the object, and the hand-object relationship. We remark again that “unknown object” means that the model of the object is not used at all in the manipulation procedure.

The aim of the proposed approach is, after performing a FC grasp of an object, to iteratively determine the movements (sequences of hand configurations) to improve a manipulation index according to the mentioned goals. The initial grasp could be non-optimal due to several reasons (e.g., accessibility or position uncertainty), but in any case the planning and execution of the initial grasp is outside the scope of this work.

Once the pursued goal is defined, an iterative procedure is started and in each iteration the only inputs are the tactile feedback and the kinematic configuration of the hand. The computation of the finger movements is done following a specific manipulation strategy for each of the mentioned goals (but they can be merged as described in Section 3.4), and an specific index to be minimized is defined to measure the quality of the manipulation actions. The iterative procedure ends when the corresponding index reaches a known minimum value, the index has not decreased after a predefined number of iterations, or the grasp configuration is getting close to the security limits imposed by the friction constraints.

The following assumptions are considered in this work:

- The robotic hand has tactile sensors to obtain information about the contacts with the manipulated object, and no other feedback source is available, as, for instance, visual information.
- Two fingers of the hand are used for the manipulation. These fingers perform a grasp comparable with a human grasp using the thumb and index fingers with the fingertips movements lying on plane [37]. This type of grasp limits the movement of the object to a plane but it allows different actions in every-day and industrial tasks, like, for instance, matching the orientation of two pieces to be assembled or inspecting an object [38,39].
- The manipulated objects are rigid bodies and their shape is unknown. The approach could work also for soft objects, there is not any specific constraint for it, but we did not determine in this work any limit for the acceptable softness.
- The friction coefficient is not identified during the manipulation. It is assumed to be above a minimum security value which can be roughly determined considering the object material and the rubber surface of the fingertips. In the experimentation we compute the movements using the minimum value of the friction coefficient between the material of the fingertips and the used objects, i.e., a value below the real friction coefficient.
- The finger joints have a low-level position control to make them reach the commanded positions, which is the most frequent case in a commercial hand with a closed controller. No force control is required at the level of the hand controller. The proposed approach uses the tactile measurements to generate commanded positions, thus it is actually acting as an implicit upper level force control loop.

### 2.2. Grasp Modeling

**Figure 1:**
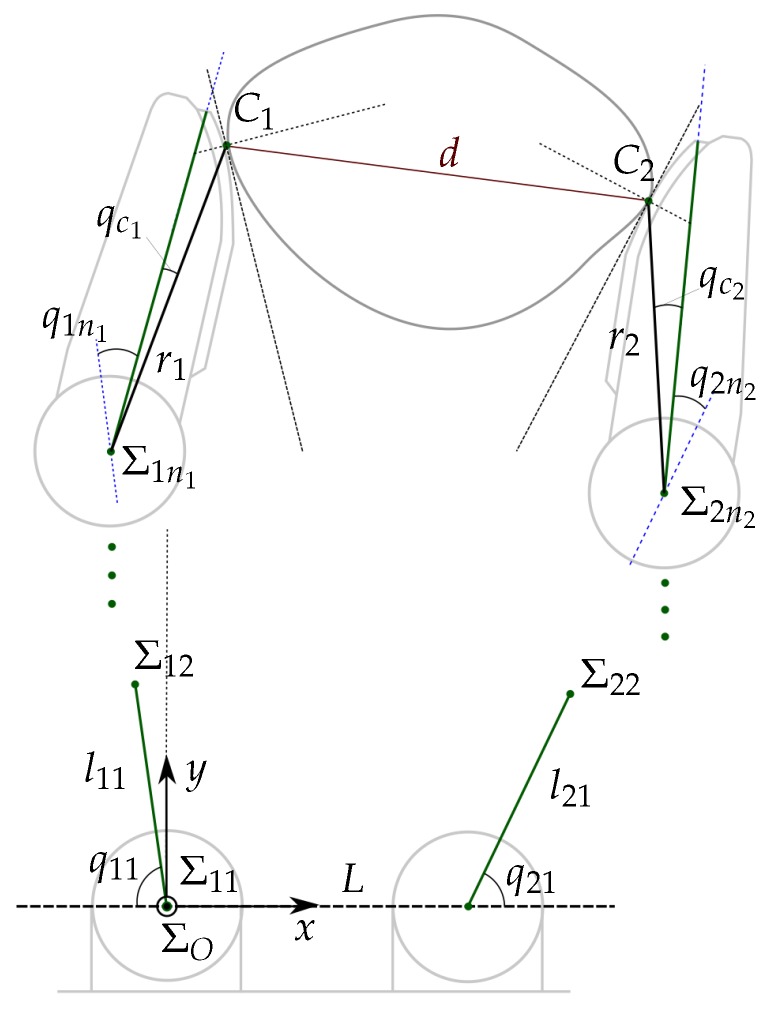
Geometric model of a two-finger grasp.

Figure 1 shows the geometric model of a two-finger grasp. A finger $f_{i}$, i∈{1,2}, is a kinematic serial chain with $n_{i}$ degrees of freedom (DOF) and $n_{i}$ links with length $l_{ij}$, $j\in \{1,\cdots ,n_{i}\}$. A joint angle $q_{ij}$ relates the position of each link to the previous one. The configuration of the finger $f_{i}$ is given by its joints angles as $q_{i}=\left\{q_{i1},\cdots ,q_{in_{i}}\right\}$. A hand configuration is given by the concatenation of the configurations of the two used fingers as $Q=\{q_{1},q_{2}\}$. Each finger link has a reference frame $\Sigma_{ij}$ fixed at its base, and the absolute reference frame $\Sigma_{O}$ is located at the base of the finger $f_{1}$.

In general, the contact between a fingertip and the object produces contact regions on the sensor pad. In this work the contact between each fingertip and the object is modeled using the punctual contact model [40]. Note that this is a consideration for the grasp modeling, since the contact on a fingertip actually may take place over a contact region which may also be composed by several disjoint subregions. For the contact model, in this work the barycenter of the actual contact region (either a single one or a set of disjoints subregions) is considered to be the current contact point. Besides, the summation of the forces sensed at each texel in the actual contact region is considered to be the current contact force applied by the finger at the equivalent punctual contact [41].

Let $C_{i}$ be the position of the contact point on finger $f_{i}$ with respect to $\Sigma_{O}$. $C_{i}$ is computed using direct kinematics of the fingers and the information provided by the tactile sensor. A virtual link is used to include the contact point information into the hand kinematics (see Figure 1). This virtual link adds a non-controllable extra DOF to each finger, which is defined by the angle $q_{c_{i}}$ or by the length $r_{i}$ of the segment between the origin $O_{\Sigma_{{in}_{i}}}$ of the reference frame $\Sigma_{{in}_{i}}$, and the contact point $C_{i}$. Then, the Euclidean distance *d* between the contact points $C_{1}$ and $C_{2}$ is given by

(1) $$d\left(C_{1},C_{2}\right)=\left|\right|\bar{C_{1}C_{2}}\left|\right|=\sqrt{{\left(C_{1_{x}}-C_{2_{x}}\right)}^{2}+{\left(C_{1_{y}}-C_{2_{y}}\right)}^{2}}$$

### 2.3. Main Manipulation Algorithm

**Figure 2:**
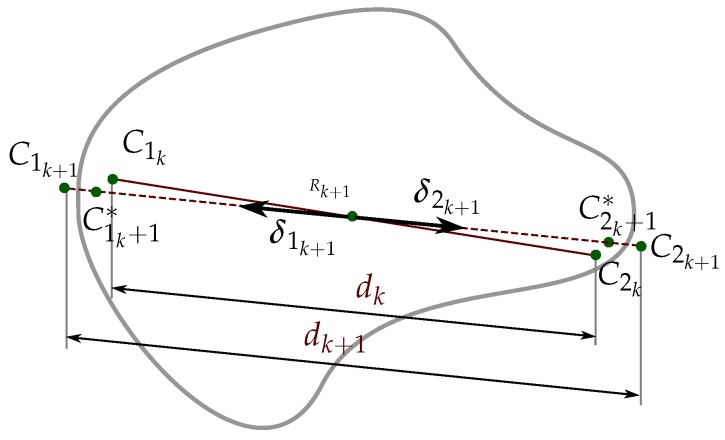
Example of the computation of $C_{i_{k+1}}$ using $C_{i_{k+1}}^{*}$, adjusting the distance $d_{k}$ to $d_{k+1}$ when the contact force $F_{k}$ is larger than $F_{\max}$.

**Figure 3:**
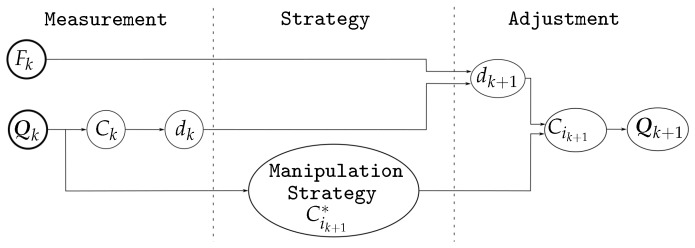
Relation between the measured variables, the role of the manipulation strategy, and the final adjustment to obtain the new hand configuration.

Algorithm 1 shows the main manipulation procedure, which is general and valid for any manipulation strategy. As inputs, the user selects the desired contact force $F_{d}$ and the manipulation strategy (MS) to pursue one of the three manipulation goals mentioned in Section 1 or a combination of them. The manipulation process stars with a blind grasp of the object, closing the fingers along a predefined path until $F_{d}$ is reached and the object has been securely grasped (lines 2 to 4). Then, the object is manipulated with an iterative procedure following the selected manipulation strategy. Each iteration *k* involves the following parts:

1. Computation of the relevant variables of the current grasp state (lines 6 to 7). $C_{1_{k}}$ and $C_{2_{k}}$ are obtained using the hand kinematics and the tactile information, and the magnitude of the grasping force $F_{k}$ is obtained as the average of the contact forces $F_{1_{k}}$ and $F_{2_{k}}$ measured on each fingertip. Although $F_{1_{k}}$ and $F_{2_{k}}$ should have the same magnitude and opposite direction, the use of the average of both measured contact forces minimizes potential measurement errors, thus
  (2) $$F_{k}=\frac{F_{1_{k}}+F_{2_{k}}}{2}$$
2. Computation of two virtual contact points $C_{1_{k+1}}^{*}$ and $C_{2_{k+1}}^{*}$ (line 8). These points are such that the movements of the fingers to make them be the new contact points changes the grasp towards the selected goal. The computation of $C_{1_{k+1}}^{*}$ and $C_{2_{k+1}}^{*}$ from $C_{1_{k}}$ and $C_{2_{k}}$ according to each manipulation strategy MS are detailed in the next section.
3. Computation of the new hand configuration $Q_{k+1}=\left\{q_{1_{k+1}},q_{2_{k+1}}\right\}$ (lines 10 to 12). Since the shape of the object is unknown, any movement of the fingers may alter the contact force $F_{k}$ allowing potential damage of the object or the hand if it increases or allowing a potential fall of the object if it decreases. In order to reduce the error $e=F_{k}-F_{d}$, the distance $d_{k}$ is adjusted in each iteration as
  (3) $$d_{k+1}=d_{k}+\Delta d$$
  with
  (4) $$\Delta d=\left\{\begin{array}{cc} f_{1}\left(e\right) & \;\mathrm{if}\;e\leq 0\\ f_{2}\left(e\right) & \;\mathrm{if}\;e>0 \end{array}\right.$$
  where $f_{1}\left(e\right)$ and $f_{2}\left(e\right)$ are user defined functions. In this work, we use $f_{1}\left(e\right)=2\lambda \left(e+e^{2}\right)$ and $f_{2}\left(e\right)=\lambda e$, with λ being a predefined constant. The reason for this is that a potential fall of the object ($F_{k}\to 0$) is considered more critical that a potential application of large grasping forces ($F_{k}\gg F_{d}$), and therefore $f_{1}\left(e\right)$ has larger gain, specially for large |e|.
**Algorithm 1:** Tactile Manipulation
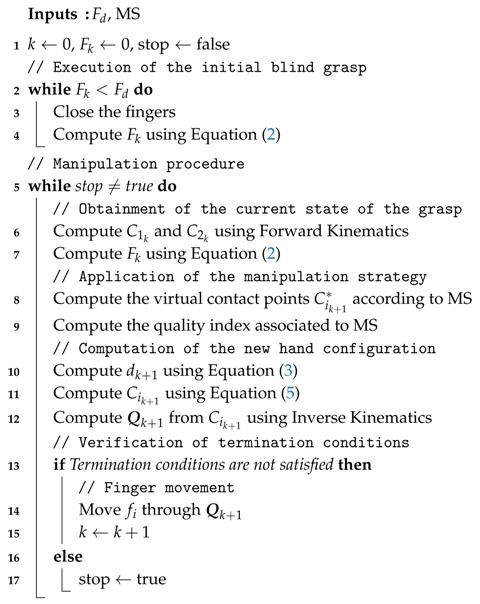
  Now, $C_{1_{k+1}}^{*}$ and $C_{2_{k+1}}^{*}$ are adjusted along the line they define to obtain the actual target contact points $C_{1_{k+1}}$ and $C_{2_{k+1}}$ at a distance $d_{k+1}$,
  (5) $$C_{i_{k+1}}=R_{k+1}+\frac{d_{k+1}}{2}\delta_{i_{k+1}},\;\;\;i\in \left\{1,2\right\}$$
  where $R_{k+1}$ is the central point between $C_{1_{k+1}}^{*}$ and $C_{2_{k+1}}^{*}$ and $\delta_{i_{k+1}}$ is the unitary vector from $R_{k+1}$ to $C_{i_{k+1}}^{*}$ (see Figure 2).
  Finally, using the inverse kinematics of the fingers, from the points $C_{1_{k+1}}$ and $C_{2_{k+1}}$ it is possible to obtain the corresponding hand configuration $Q_{k+1}=\left\{q_{1_{k+1}},q_{2_{k+1}}\right\}$.
  Figure 3 illustrates the relationship between the measured variables, the role played by the manipulation strategy in the computation of the auxiliary variables $C_{i_{k+1}}^{*}$, and the variables involved in the final adjustment to obtain the new hand configuration (with independence of the manipulation strategy).
4. Termination conditions (line 13). The iterative manipulation procedure is applied until any of the following four stop conditions is activated, two of them associated with the quality index and the other two with the motion constraints:
  - The quality index reaches the optimal value.
  - The current optimal value of the quality index is not improved during a predetermined number of iterations. Note that the index may not be improved monotonically, it could may become worst or oscillate alternating small improvements and worsening.
  - The expected grasp at the computed contact points does not satisfy the friction constraints.
  - The computed contact points do not belong to the workspace of the fingers. This condition is activated when the computed target contact points $C_{1_{k+1}}$ and $C_{2_{k+1}}$ are not reachable by the fingers, i.e., $Q_{k+1}=\left\{q_{1_{k+1}},q_{2_{k+1}}\right\}$ does not lie within the hand workspace.
5. Finger movements (line 14). When none of the termination conditions is activated, the hand is moved towards $Q_{k+1}$ to make the fingers reach the desired target contact points $C_{1_{k+1}}$ and $C_{2_{k+1}}$. After the finger movements a new manipulation iteration begins.

## 3. Manipulation Strategies

This section presents the manipulation strategies to optimize the hand configuration, the grasp quality and the object orientation, or a combination of them, according to a desired goal using only information from the current hand configuration and from the tactile sensors. The following subsections introduce the index to be optimized and the procedure to generate the two virtual contact points $C_{1_{k+1}}^{*}$ and $C_{2_{k+1}}^{*}$ for each manipulation strategy and for a combination of them.

### 3.1. Optimizing the Hand Configuration

#### 3.1.1. Index to be Optimized

The optimization of the hand configuration implies that the fingers must try to reach specific positions while preventing the fall of the object. These positions are generally defined by the middle-range positions of the joints, but it could be also arbitrarily defined by the user according to the particular features of the used hand (in the middle-range positions the joints are far away from their mechanical limits, thus there is a potential wider range of movements).

Let $q_{0_{ij}}$ be the predefined desired specific position of the *j*-th joint of the finger *i*, then $Q_{0}=\left\{q_{0_{ij}},i\in \left\{1,2\right\},j\in \left\{1,n_{i}\right\}\right\}$ is the desired specific configuration of the hand. Then, the goodness of the hand configuration is indicated by a quality index $I_{\mathrm{hc}}$ computed according to the current joint values $q_{ij}$ as

(6) $$I_{\mathrm{hc}}=\sum_{i=1}^{2}\sum_{j=1}^{n_{i}}{\left(\frac{q_{ij}-q_{0_{ij}}}{q_{\max_{ij}}-q_{\min_{ij}}}\right)}^{2}$$

where $q_{\max_{ij}}$ and $q_{\min_{ij}}$ are the maximum and minimum limits of the *j*-th joint of the finger *i*, respectively. The hand configuration is improved by minimizing $I_{\mathrm{hc}}$, which favors the hand configurations with the joints as close as possible to the desired specific positions [42].

#### 3.1.2. Optimization Strategy

In this case, the goal configuration of the hand is known with independence of the object shape, thus it is trivial to move the hand towards it, the key point is to do it allowing an adequate adjustment of the distance $d_{k}$ between the contact points in each iteration to prevent the object from falling. Then, the hand configuration is updated in each iteration as

(7) $$Q_{k+1}^{*}=Q_{k}+\Delta Q$$

where

(8) $$\Delta Q=\eta (Q_{0}-Q_{k})$$

is a small enough vector pointing from the current configuration $Q_{k}=\left\{q_{1_{k}},q_{2_{k}}\right\}$ to $Q_{0}$, i.e., η must be chosen to properly fix the advance of the hand configuration in each iteration. As a practical approach, when the angles are measured in degrees, ΔQ≤1 was found to work well, this is achieved with

(9) $$\eta =\frac{\tanh(||Q_{0}-Q_{k}||)}{\left|\right|Q_{0}-Q_{k}\left|\right|}$$

where tanh is used to bound η when the current configuration of the hand $Q_{k}$ is far from $Q_{0}$. From Equations (8) and (9) results

(10) $$\Delta Q=\frac{\tanh(||Q_{0}-Q_{k}||)}{\left|\right|Q_{0}-Q_{k}\left|\right|}\left(Q_{0}-Q_{k}\right)$$

Finally, from $Q_{k+1}^{*}$ it is straightforward to obtain the virtual contact points $C_{1_{k+1}}^{*}$ and $C_{2_{k+1}}^{*}$ using the direct kinematics of the hand.

**Figure 4:**
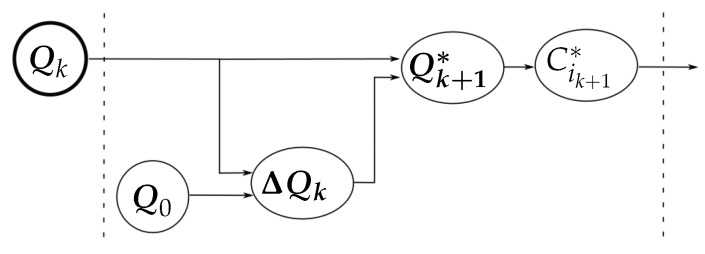
Variables involved in the optimization of the hand configuration.

Figure 4 summarizes the relation between the variables involved in the computation of $C_{1_{k+1}}^{*}$ and $C_{2_{k+1}}^{*}$ for the optimization of the hand configuration (according to the general diagram shown in Figure 3).

### 3.2. Optimizing the Grasp Quality

#### 3.2.1. Index to be Optimized

**Figure 5:**
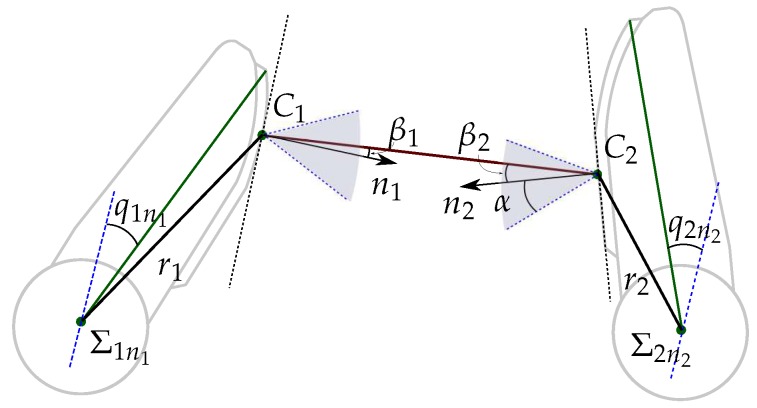
Fingertips and angles used to compute the friction constraints.

The optimization of the grasp quality implies that the fingers must manipulate the object increasing the security margin of the force-closure grasp given by the angles $\beta_{i}$, i∈{1,2} (see Figure 5). i.e., the segment connecting both contact points must lie far from the boundary of the friction cones. Then, the grasp quality is measured using a quality index $I_{\mathrm{gq}}$ based on the angles $\beta_{i}$ as

(11) $$I_{\mathrm{gq}}=\frac{1}{2}\sum_{i=1}^{2}\left|\beta_{i}\right|$$

Thus, the grasp quality is improved by minimizing $I_{\mathrm{gq}}$.

#### 3.2.2. Optimization Strategy

Using basic geometry and the information obtained from the tactile sensors and the finger kinematics, the angles $\beta_{i}$ can be computed as functions of the current contact points $C_{i}$, the origin $O_{\Sigma_{in_{i}}}$ of the reference frame $\Sigma_{in_{i}}$, and the length $r_{i}$ and the joint angle $q_{c_{i}}$ of the virtual link at the fingertips (all the variables are computed for the iteration *k*, thus, to improve legibility, subindex *k* have been removed),

(12) $$\beta_{1}=\arccos\left(\frac{-|\bar{O_{\Sigma_{in_{i}}}C_{2}}{|}^{2}+r_{1}^{2}+{\left|\bar{C_{1}C_{2}}\right|}^{2}}{2r_{1}\left|\bar{C_{1}C_{2}}\right|}\right)+q_{c_{1}}-\pi$$

(13) $$\beta_{2}=\arccos\left(\frac{-|\bar{O_{\Sigma_{in_{i}}}C_{1}}{|}^{2}+r_{2}^{2}+{\left|\bar{C_{1}C_{2}}\right|}^{2}}{2r_{2}\left|\bar{C_{1}C_{2}}\right|}\right)+q_{c_{2}}-\pi$$

The gradient of $\beta_{i}\left(Q_{k}\right)$ at the current configuration of the hand, $\nabla \beta_{i}\left(Q_{k}\right)$, is used to compute the next virtual configuration of the hand $Q_{k+1}^{*}$ as

(14) $$Q_{k+1}^{*}=Q_{k}+\Delta Q$$

where ΔQ is now given by

(15) $$\Delta Q=\frac{1}{2}\tanh\left(\beta_{1}\right)\frac{\nabla \beta_{1}}{||\nabla \beta_{1}\left|\right|}+\frac{1}{2}\tanh\left(\beta_{2}\right)\frac{\nabla \beta_{2}}{||\nabla \beta_{2}\left|\right|}$$

Finally, as in the previous strategy, from $Q_{k+1}^{*}$ it is straightforward to obtain the virtual contact points $C_{i_{k+1}}^{*}$ and $C_{i_{k+1}}^{*}$ using the direct kinematics of the hand.

**Figure 6:**
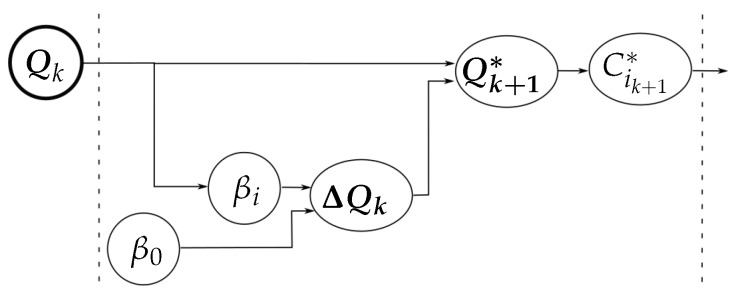
Variables involved in the optimization of the grasp quality.

Figure 6 summarizes the relation between the variables involved in the computation of $C_{1_{k+1}}^{*}$ and $C_{2_{k+1}}^{*}$ for the optimization of the grasp quality (according to the general diagram shown in Figure 3).

### 3.3. Optimizing the Object Orientation

#### 3.3.1. Index to be Optimized

The optimization of the object orientation implies that the fingers must rotate the object towards a desired goal orientation. The orientation of the object in the initial blind grasp is considered as $\gamma_{0}=0$, and therefore the desired orientation of the object $\gamma_{d}$ is relative to it. Then, the manipulation strategy must reduce the difference between $\gamma_{d}$ and the current object orientation $\gamma_{k}$. The quality index could be just the orientation error $|\gamma_{d}-\gamma_{k}|$, but in order to constrain it to the range [0,1] it is normalized dividing by $\gamma_{i}-\gamma_{d}$, $\gamma_{i}$ being the current orientation at the time $\gamma_{d}$ is given, i.e.,

(16) $$I_{\mathrm{oe}}=\left|\frac{\gamma_{d}-\gamma_{k}}{\gamma_{d}-\gamma_{i}}\right|$$

#### 3.3.2. Optimization Strategy

The orientation of the object $\gamma_{k}$ can be computed using basic geometry and the information obtained from the tactile sensors and the finger kinematics, no other external feedback is considered (like, for instance, a vision system) although it could exist at a higher level (for instance to determine $\gamma_{d}$, but this is outside of the scope of this work). For fingertips with circular shape, the current object orientation $\gamma_{k}$ is given by [43]

(17) $$\begin{array}{ccc} \gamma_{k} & = & \frac{2R+d_{k}}{d_{k}}\left(\theta_{0}-\theta \right)+\\ & & \frac{R}{d_{k}}\sum_{j=1}^{n_{1}}\left(q_{1j_{\gamma_{0}}}-q_{1j_{k}}\right)-\sum_{j=1}^{n_{2}}\left(q_{2j_{\gamma_{0}}}-q_{2j_{k}}\right) \end{array}$$

being

θ the average of the two angles between an arbitrary reference axis attached to the object and the directions normal to each fingertip at the corresponding contact point,
$\theta_{0}$ the value of θ at the initial grasp (i.e., for $\gamma_{0}$),
$q_{ij_{k}}$ the current value of the ij-th joint (i.e., joint $j=1,\ldots ,n_{i}$ of finger i=1,2),
$q_{ij_{\gamma_{0}}}$ the value of the ij-th joint at the initial grasp (i.e., for $\gamma_{0}$),
$d_{k}$ the distance between the contact points, and
*R*  the radius of the fingertip.

The first term in Equation (17) has a factor that depends on the variation of θ, then, since θ does not change significantly during the manipulation (i.e., $\theta \approx \theta_{0}$) the first term can be neglected. Thus, $\gamma_{k}$ can be approximated by

(18) $$\gamma_{k}\approx \frac{R}{d_{k}}\sum_{j=1}^{n_{1}}\left(q_{1j_{\gamma_{0}}}-q_{1j_{k}}\right)-\sum_{j=1}^{n_{2}}\left(q_{2j_{\gamma_{0}}}-q_{2j_{k}}\right)$$

Since the finger movements are small and $\gamma_{k}$ is recomputed in each iteration, this approximation is accurate enough for the manipulation goal.

**Figure 7:**
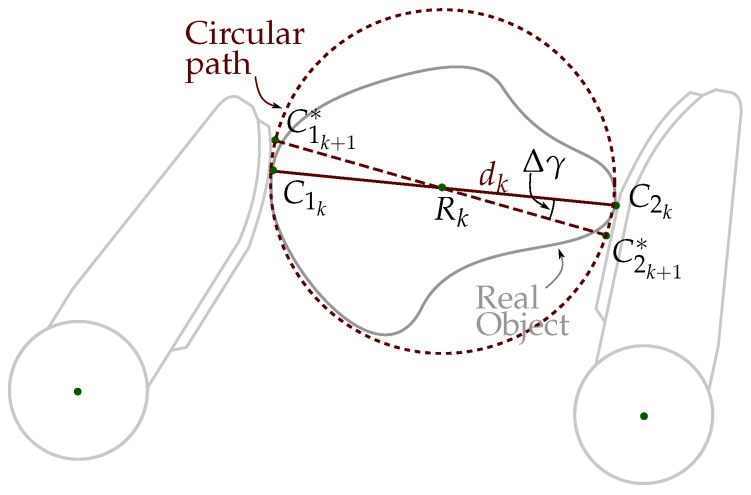
Movements used for the optimization of the object orientation. $C_{1_{k+1}}$ and $C_{2_{k+1}}$ are computed over a circular path with diameter $d_{k}$ centered at $R_{k}$.

Now, the virtual contact points $C_{1_{k+1}}^{*}$ and $C_{2_{k+1}}^{*}$ are computed considering that the fingers are moved to produce the displacement of the contact points on the sensor pad along a circular path given by (see Figure 7):

(19) $$\begin{array}{ccc} C_{1_{{k+1}_{x}}}^{*} & = & R_{k_{x}}-\left(d_{k}/2\right)\cos\left(\gamma_{k+1}\right) \end{array}$$

(20) $$\begin{array}{ccc} C_{1_{{k+1}_{y}}}^{*} & = & R_{k_{y}}-\left(d_{k}/2\right)\sin\left(\gamma_{k+1}\right) \end{array}$$

(21) $$\begin{array}{ccc} C_{2_{{k+1}_{x}}}^{*} & = & R_{k_{x}}+\left(d_{k}/2\right)\cos\left(\gamma_{k+1}\right) \end{array}$$

(22) $$\begin{array}{ccc} C_{2_{{k+1}_{y}}}^{*} & = & R_{k_{y}}+\left(d_{k}/2\right)\sin\left(\gamma_{k+1}\right) \end{array}$$

i.e., the new virtual positions are points on a circumference with diameter $d_{k}$ centered at the middle point, $R_{k}$, between the points $C_{1_{k}}$ and $C_{2_{k}}$, and

(23) $$\gamma_{k+1}=\gamma_{k}+\tanh\left(\gamma_{k}\right)\Delta \gamma$$

Δγ is chosen empirically and small enough to assure small movements of the object in each manipulation step.

Note that in this case it was not necessary to compute $Q_{k+1}$ as an intermediate step to determine the virtual contact points $C_{i_{k+1}}^{*}$. Instead, now $Q_{k+1}$ can be deduced from $C_{i_{k+1}}^{*}$ applying inverse kinematic. This is relevant since the direction of $\Delta Q=Q_{k+1}^{*}-Q_{k}$ is necessary to combine different manipulation strategies, as will be shown in Section 3.4.

**Figure 8:**
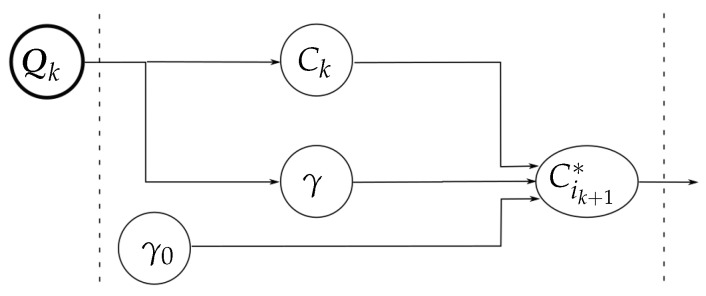
Variables involved in the optimization of the object orientation.

Figure 8 summarizes the relation between the variables involved in the computation of $C_{1_{k+1}}^{*}$ and $C_{2_{k+1}}^{*}$ for the optimization of the object orientation (according to the general diagram shown in Figure 3).

### 3.4. Combining Manipulation Strategies

#### 3.4.1. Index to be Optimized

The approach allows the combination of two or more manipulation strategies, for this purpose a combined quality index $I_{\mathrm{cq}}$ is computed as a lineal combination of the quality indexes associated to the combined manipulation strategies, i.e.,

(24) $$I_{\mathrm{cq}}=\sum_{j}\omega_{j}I_{j}$$

where $\omega_{j}>0$ are weighting coefficients.

#### 3.4.2. Optimization Strategy

When two or more manipulation strategies are combined, the target configuration of the hand $Q_{k+1}$ is computed as the current hand configuration plus a lineal combination of the incremental movements $\Delta Q_{j}$ obtained by each manipulation strategy *j* individually, i.e.,

(25) $$Q_{k+1}^{*}=Q_{k}+\sum_{j}\omega_{j}\Delta Q_{j}$$

with $\omega_{j}>0$ satisfying $\sum_{j}\omega_{j}=1$ to avoid unexpected large movements. The coefficients $\omega_{j}$ can be arbitrarily adjusted to give different weights to each combined strategy. It must be remarked that the final movement determined to optimize the combined index does not imply the individual optimization of all the involved individual indexes.

Then, from $Q_{k+1}^{*}$ it is straightforward to obtain the virtual contact points $C_{1_{k+1}}^{*}$ and $C_{2_{k+1}}^{*}$ using the direct kinematics of the hand.

## 4. Experimental Validation

The proposed approach has been fully implemented using C++. The system setup and some examples of experimental results are presented below to illustrate the performance of the approach.

### 4.1. System Setup

**Figure 9:**
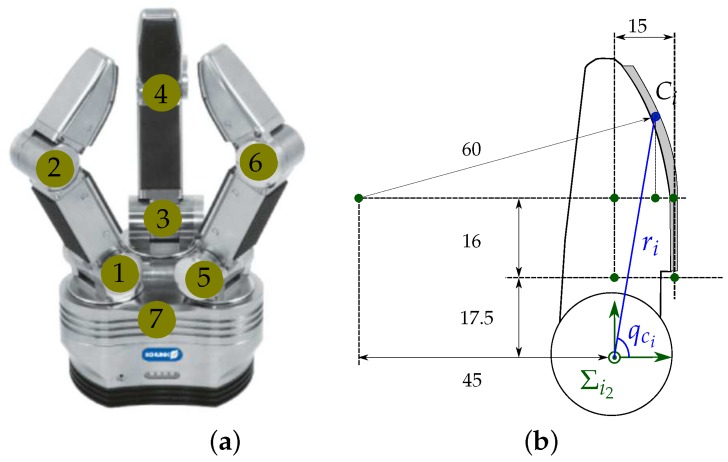
(**a**) Schunk Dexterous Hand (SDH2) with joints labels; (**b**) lateral view of the fingertip with the sensor pad (distances are in millimeters).

**Figure 10:**
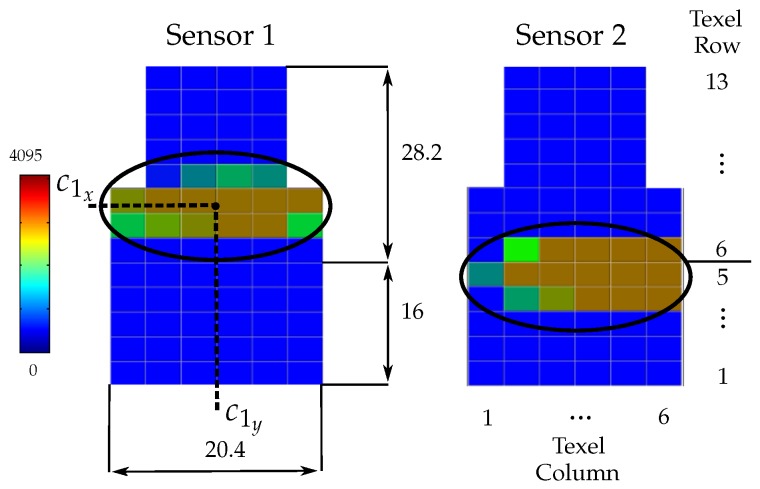
Graphical representation of tactile measurements highlighting with ellipses the contact region on each sensor pad. The bar in the left indicates the scale of colors corresponding to the force values returned by each texel, the range of returned values from 0 to 4095 corresponds to a range of forces from 0 to 3 N. The five lower rows of texels correspond to the planar part of the sensor. All the lengths are in millimeters. (see also Figure 9c).

The Schunk Dexterous Hand (SDH2) shown in Figure 9a was used for the experimental validation. This is a three-finger hand, each finger has two DOF and another one allows the rotation of two fingers around their bases to work opposite to each other, making a total of seven DOF. The SDH2 has tactile sensors on the surface of the proximal and distal phalanges. A detailed description of the hand kinematics is presented in [44]. In this work, only the fingertips of the two fingers working opposed to each other are used for the manipulation. The sensor surface on the fingertips is composed of a planar part with length 16 mm and a curve part with radius 60 mm (Figure 9b). The planar part of the sensor pad includes the rows of texels 1 to 5, and the curved part the rows of texels 6 to 13; the wide of the sensor is 6 texels in the lower part and 4 texels in the upper part, making a total of 68 sensitive texels (Figure 10). Each texel of the sensor pads returns a value from 0, when no pressure is applied, to 4095, for a maximum measurable normal force per texel of 3 N. As stated in Section 2.2, we consider the barycenter of the contact region as the current contact point between the object and the fingertip and the summation of the forces over all the texels in the contact region as the current contact force [41] (see Figure 10). It must be noted that when the contact is produced only on one or two texels the measured force is limited to up to 3 or 6 N respectively and these cases must be specially considered to avoid pushing the fingers trying to get larger forces. Besides, since the tactile sensors do not provide tangential components of the grasping forces, in the experiments the actual contact force could be larger than the measured one, which is not a significant problem, unless extremely fragile objects are manipulated and the normal forces are quite close to the maximal tolerated forces. There are proposals of tactile sensing devices that allow the measurement of the real applied forces [45]. Nevertheless, since the proposed approach also considers the angles $\beta_{i}$ between the normal directions at the contact points and the force direction (defined by the contact points), the explicit measurement of the tangential force component is not necessary for the computation of the grasp security margin.

### 4.2. Experimental Results

In the following illustrative examples the fingers are blindly closed around an unknown object until the measured grasping force reaches an arbitrary desired value $F_{d}=5$ N. This force value was chosen considering the range of the tactile sensors, the forces the hand can apply and that the manipulated objects were hard rigid bodies. The objects used for the experiments were selected looking for different object shapes (with small and large curvatures) and different object boundaries (smooth and irregular), so the performance of the proposed approach can be illustrated under different conditions. The initial position of the object varies in each execution of the experiments and therefore the initial grasp configuration and the initial contact points are unknown a priori by the system. The friction coefficient considered in the calculations was μ=0.4 (friction cone angle of only α=21.8 degrees), which is below the expected real physical value. The constant λ to adjust the distance between the contact points according to Equation (4) was set to λ=0.25 mm. Videos of experimental executions can be found in http://goo.gl/ivFd0q.

**Figure 11:**
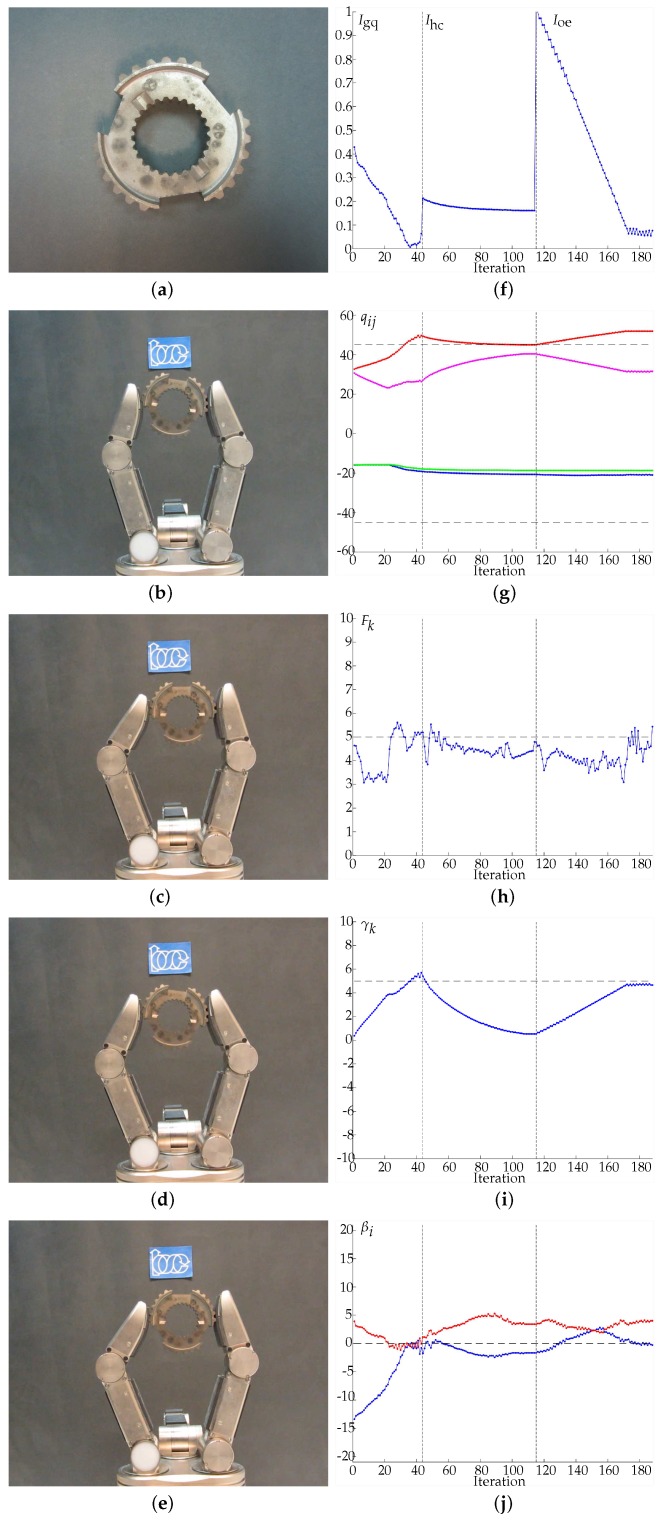
Example 1. (**a**) Manipulated object. (**b**) Initial grasp. (**c**) Hand configuration after optimizing $I_{\mathrm{gq}}$. (**d**) Hand configuration after optimizing $I_{\mathrm{hc}}$. (**e**) Hand configuration after optimizing $I_{\mathrm{oe}}$. (**f**) Evolution of the quality indexes. The manipulations improving $I_{\mathrm{gq}}$, $I_{\mathrm{hc}}$ and $I_{\mathrm{oe}}$ ended after 3.864 s and 43 iterations, 4.486 s and 70 iterations and 9.083 s and 73 iterations, respectively. (**g**) Evolution of the joints values in degrees, $q_{11}$ in blue, $q_{12}$ in red, $q_{21}$ in green, $q_{22}$ in magenta. (**h**) Average force $F_{k}$ in Newtons, the dashed line indicates $F_{d}$. (**i**) Evolution of the object orientation in degrees. (**j**) Angles $\beta_{i}$ in degrees, $\beta_{1}$ in blue and $\beta_{2}$ in red (the dashed line indicates the optimal value of $\beta_{i}$). Note that the non-smooth and toothed surface of the manipulated object produces more than one contact region on each fingertip without generating any manipulation problem.

**Figure 12:**
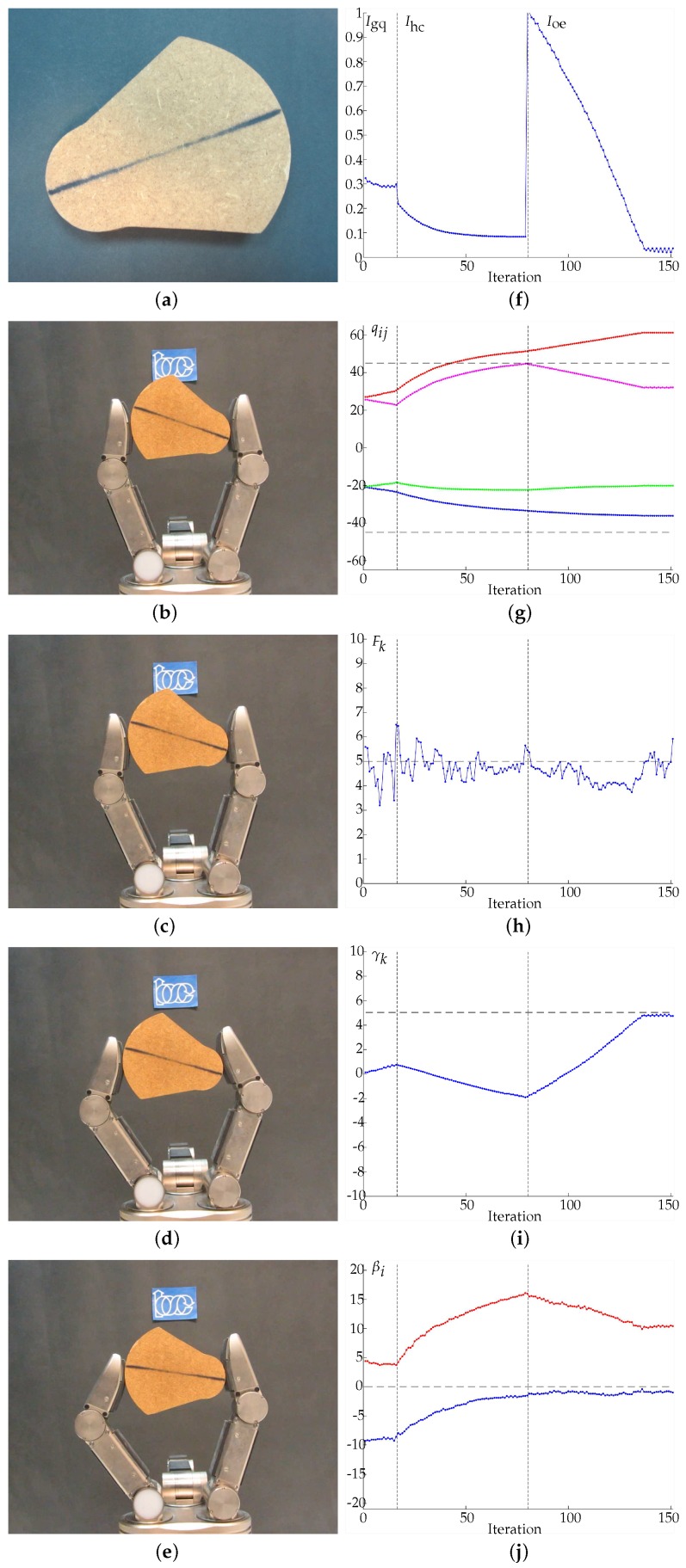
Example 2. (**a**) Manipulated object. (**b**) Initial grasp. (**c**) Hand configuration after optimizing $I_{\mathrm{gq}}$. (**d**) Hand configuration after optimizing $I_{\mathrm{hc}}$. (**e**) Hand configuration after optimizing $I_{\mathrm{oe}}$. (**f**) Evolution of the quality indexes. The manipulations improving $I_{\mathrm{gq}}$, $I_{\mathrm{hc}}$ and $I_{\mathrm{oe}}$ ended after 1.171 s and 16 iterations, 4.687 s and 62 iterations and 9.709 s and 71 iterations, respectively. (**g**) Evolution of the joints values in degrees, $q_{11}$ in blue, $q_{12}$ in red, $q_{21}$ in green, $q_{22}$ in magenta. (**h**) Average force $F_{k}$ in Newtons, the dashed line indicates $F_{d}$. (**i**) Evolution of the object orientation in degrees. (**j**) Angles $\beta_{i}$ in degrees, $\beta_{1}$ in blue and $\beta_{2}$ in red (the dashed line indicates the optimal value of $\beta_{i}$).

**Figure 13:**
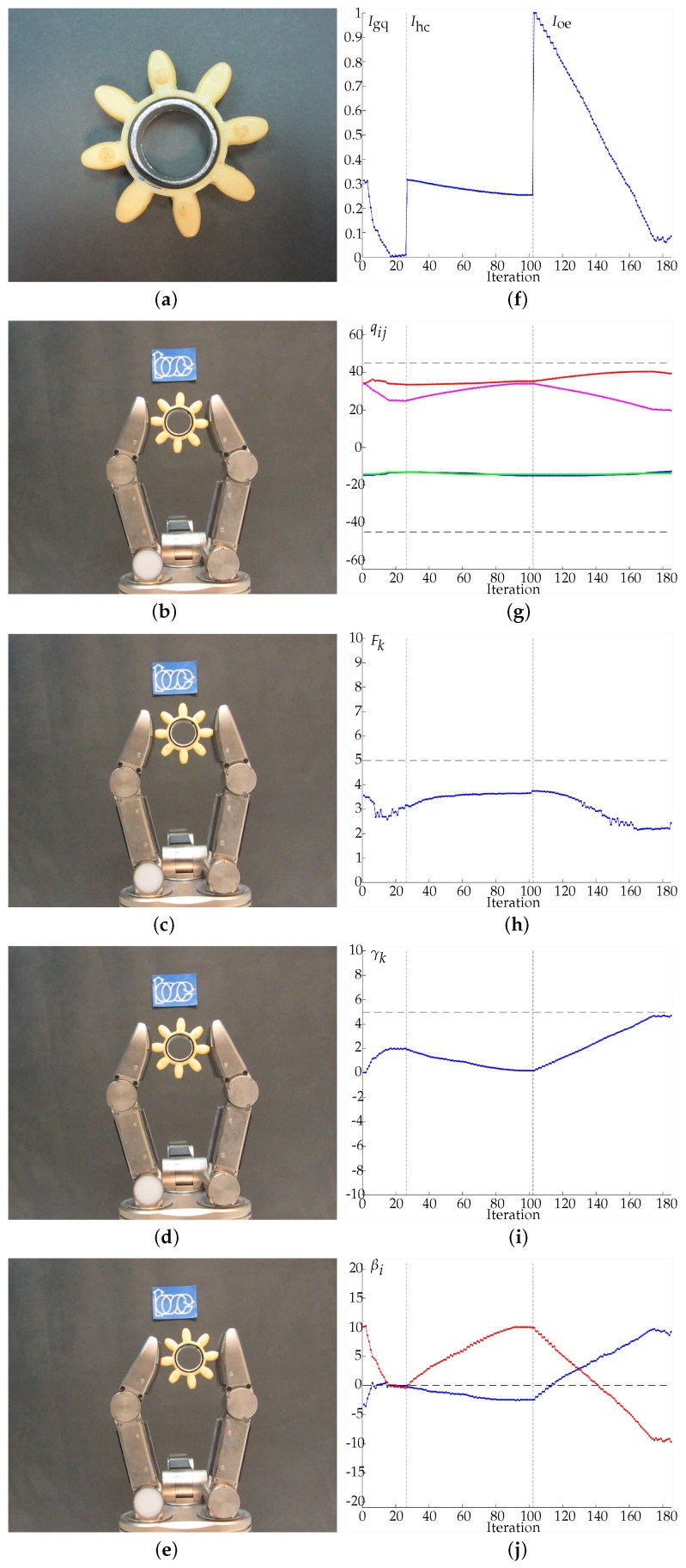
Example 3. (**a**) Manipulated object. (**b**) Initial grasp. (**c**) Hand configuration after optimizing $I_{\mathrm{gq}}$. (**d**) Hand configuration after optimizing $I_{\mathrm{hc}}$. (**e**) Hand configuration after optimizing $I_{\mathrm{oe}}$. (**f**) Evolution of the quality indexes. The manipulations improving $I_{\mathrm{gq}}$, $I_{\mathrm{hc}}$ and $I_{\mathrm{oe}}$ ended after 2.439 s and 26 iterations, 4.627 s and 75 iterations and 8.779 s and 82 iterations, respectively. (**g**) Evolution of the joints values in degrees, $q_{11}$ in blue, $q_{12}$ in red, $q_{21}$ in green, $q_{22}$ in magenta. (**h**) Average force $F_{k}$ in Newtons, the dashed line indicates $F_{d}$. (**i**) Evolution of the object orientation in degrees. (**j**) Angles $\beta_{i}$ in degrees, $\beta_{1}$ in blue and $\beta_{2}$ in red (the dashed line indicates the optimal value of $\beta_{i}$). Note that, due to the shape of the manipulated object, the contact is produced on a limited region of the sensor and therefore the force $F_{k}$ cannot reach the desired force $F_{d}$.

**Figure 14:**
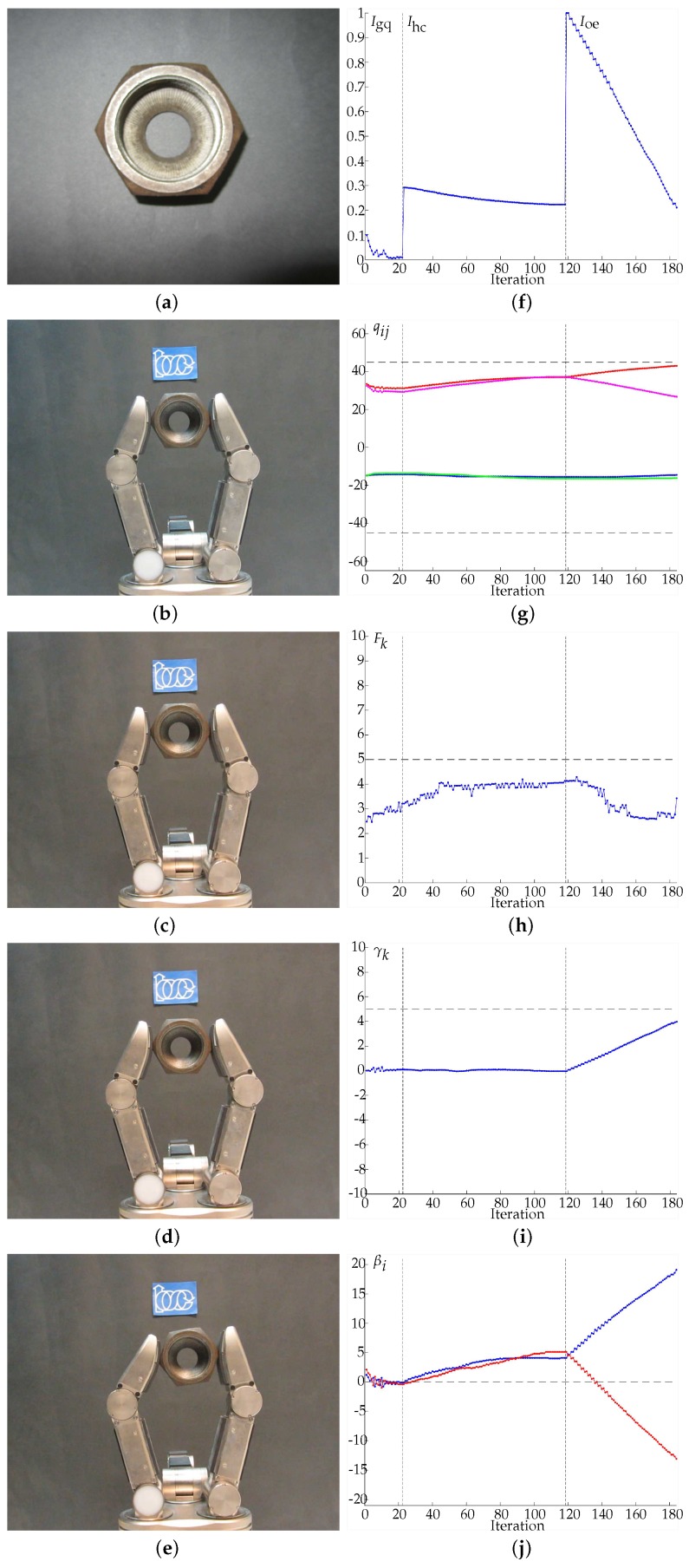
Example 4. (**a**) Manipulated object. (**b**) Initial grasp. (**c**) Hand configuration after optimizing $I_{\mathrm{gq}}$. (**d**) Hand configuration after optimizing $I_{\mathrm{hc}}$. (**e**) Hand configuration after optimizing $I_{\mathrm{oe}}$. (**f**) Evolution of the quality indexes. The manipulations improving $I_{\mathrm{gq}}$, $I_{\mathrm{hc}}$ and $I_{\mathrm{oe}}$ ended after 2.122 s and 22 iterations, 5.558 s and 95 iterations and 5.347 s and 65 iterations, respectively. (**g**) Evolution of the joints values in degrees, $q_{11}$ in blue, $q_{12}$ in red, $q_{21}$ in green, $q_{22}$ in magenta. (**h**) Average force $F_{k}$ in Newtons, the dashed line indicates $F_{d}$. (**i**) Evolution of the object orientation in degrees. (**j**) Angles $\beta_{i}$ in degrees, $\beta_{1}$ in blue and $\beta_{2}$ in red (the dashed line indicates the optimal value of $\beta_{i}$). Note that as in Example 3 the contact region is quite small due the object shape and therefore the force $F_{k}$ cannot reach the desired force. The manipulation ended without reaching the desired object orientation because the friction constraints were not satisfied and the object could slip out of the hand.

In Examples 1 to 4 (Figure 11, Figure 12, Figure 13 and Figure 14, respectively) four different objects are manipulated improving the three quality indexes sequentially, first the manipulation optimizes $I_{\mathrm{gq}}$, then $I_{\mathrm{hc}}$ and finally $I_{\mathrm{oe}}$. When $I_{\mathrm{gq}}$ is improved, the angles $\beta_{i}$ are minimized according to the expected behavior of the manipulation strategy. For the improvement of $I_{\mathrm{hc}}$, $Q_{0}=\left\{-45,45,-45,45\right\}$ is considered as the desired hand configuration. Finally, for the improvement of $I_{\mathrm{oe}}$, the desired goal is an object rotation of 5 degrees clockwise. On the sub-figures showing charting results, a vertical dotted line is depicted to highlight the iterations when the optimization index changes. Particular details of each experiment are given in the caption of each figure.

**Figure 15:**
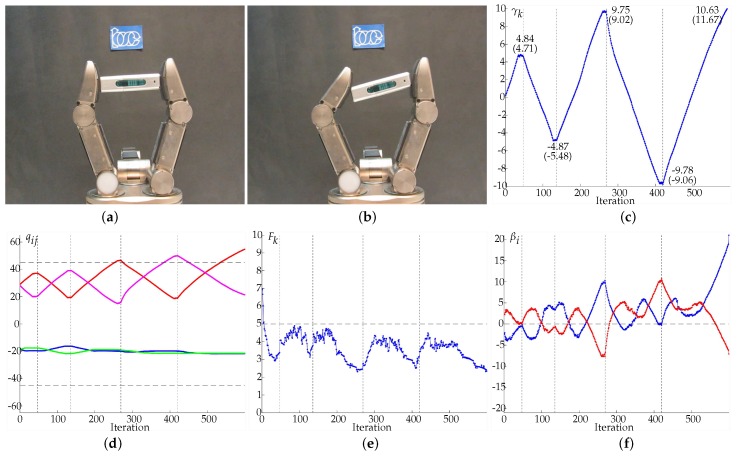
Example 5. (**a**) Initial grasp. (**b**) Final grasp. (**c**) Evolution of the object orientation $\gamma_{k}$ with sequential setpoints 5, −5, 10, −10 and 15 degrees. (**d**) Evolution of the joints values in degrees, $q_{11}$ in blue, $q_{12}$ in red, $q_{21}$ in green, $q_{22}$ in magenta. (**e**) Average force $F_{k}$ in Newtons, the dashed line indicates $F_{d}$. (**f**) Angles $\beta_{i}$ in degrees, $\beta_{1}$ in blue and $\beta_{2}$ in red (the dashed line indicates the optimal value of $\beta_{i}$).

In Example 5 (Figure 15) the object was successively rotated clockwise and counterclockwise with desired orientations $\gamma_{d}$ set to 5, −5, 10, −10, and 15 degrees. The change of setpoint was manually done once the system has activated a termination condition for the current setpoint. In the first four cases the termination condition was the arrival of $I_{\mathrm{oe}}$ to the expected value according to the system internal measurements, i.e., $\gamma_{k}\approx \gamma_{d}$ (see Figure 15c), and in the last case the manipulation ended because the expected next value of the angle $\beta_{1}$ exceeded the friction cone limit before arriving to $\gamma_{d}=15$ degrees (see the evolution of $\beta_{1}$ in Figure 15f), meaning that there was a risk of sliding and the object could flip away from the hand. The real orientations of the object when the terminal conditions were activated, measured by an external vision system, are given in Figure 15c in parenthesis below the corresponding values obtained from internal measurements. Δγ was set to 0.25 degrees.

**Figure 16:**
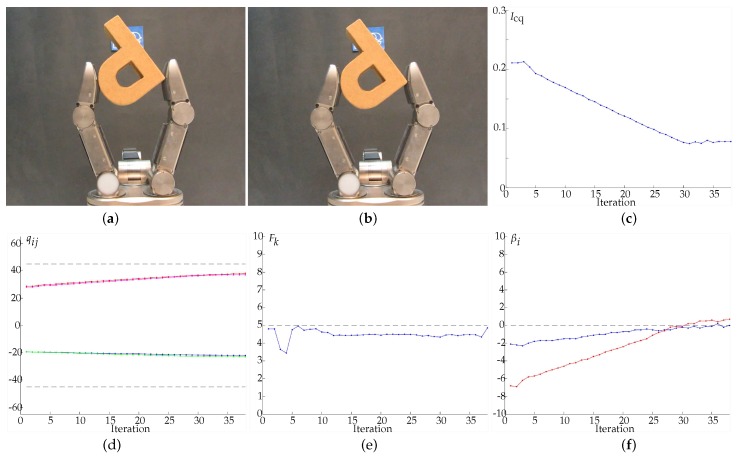
Example 6. (**a**) Initial grasp. (**b**) Final grasp. (**c**) Evolution of the index $I_{\mathrm{cq}}=0.5I_{hc}+0.5I_{gq}$. (**d**) Evolution of the joints values in degrees, $q_{11}$ in blue, $q_{12}$ in red, $q_{21}$ in green, $q_{22}$ in magenta. (**e**) Average force $F_{k}$ in Newtons, the dashed line indicates $F_{d}$. (**f**) Angles $\beta_{i}$ in degrees, $\beta_{1}$ in blue and $\beta_{2}$ in red (the dashed line indicates the optimal value of $\beta_{i}$).

In Example 6 (Figure 16) two manipulation strategies were combined, optimizing the hand configuration and the grasp quality simultaneously. The strategies were combined using $\omega_{1}=\omega_{2}=0.5$ in Equations (24) and (25), i.e., $I_{\mathrm{cq}}=0.5I_{hc}+0.5I_{gq}$. In this example $\beta_{i}$ tends to zero according to the optimization of the grasp quality while the joints tend to their desired specific positions. The manipulation ended after 2.85 s and 38 iterations because $I_{\mathrm{cq}}$ did not improve the current optimal value during 10 iterations. Note that the optimization of $I_{\mathrm{cq}}$ does not imply the optimization of $I_{hc}$ and $I_{gq}$.

## 5. Summary and Future Work

This paper has proposed an approach to manipulate unknown objects based on tactile and kinematic information, using two fingers and pursuing three common manipulation goals: the optimization of the hand configuration, the optimization of grasp quality and the optimization of the object orientation. The proposed manipulation strategies can be applied individually or in a combined way. The approach can be applied to different type of robotic hands, since the only requirements are the knowledge of the hand kinematics, a position control of the fingertips and the availability of tactile information during the manipulation. Note that, in the general case, more degrees of freedom per finger may allow a larger range of manipulation movements.

A natural extension of the proposed approach is the consideration of grasps with more than two fingers, which allow the rotation of the object around any axis. In this case, the system could be underdetermined and it would require a different strategy to adjust the modules of the forces applied by each finger, but the same basic ideas behind each of the manipulations strategies could still be applied. In this sense, note that: (a) moving the fingers to predefined specific configurations is straightforward; (b) movements that potentially improve the grasp quality could be determined if the contact points and the contact force vectors are known (even when this is not evident in the frequent case that the sensors return only the module of the normal component instead of the actual contact force); and (c) finding a (at least approximate) relation between a change in the 3D object orientation and the required finger joint movements looks as a feasible problem by replacing the movements of contact points along a circular path used in this work by movements along a path on spheres centered at some specific point of the object.

From the hardware point of view, this would require fingers with more than two DOF and not all of them producing rotations around parallel axis, in order to avoid hard constraints in the manipulation due to limitations of the joint ranges.

Another topic for future work is the use of the information about the object shape obtained while it is manipulated to optimize the following finger movements. This would help to produce more efficient and smoother movements.
